# Predictors of COVID-19 From a Statewide Digital Symptom and Risk Assessment Tool: Cross-Sectional Study

**DOI:** 10.2196/46026

**Published:** 2023-07-25

**Authors:** Benjamin L Schooley, Abdulaziz Ahmed, Justine Maxwell, Sue S Feldman

**Affiliations:** 1 Brigham Young University Provo, UT United States; 2 University of Alabama at Birmingham Birmingham, AL United States

**Keywords:** COVID-19, risk assessment, symptom tracker, passport application, surveillance, mobile app, multiple linear regression, healthcheck, public health informatics, decision support system, health information technology

## Abstract

**Background:**

Some of the most vexing issues with the COVID-19 pandemic were the inability of facilities and events, such as schools and work areas, to track symptoms to mitigate the spread of the disease. To combat these challenges, many turned to the implementation of technology. Technology solutions to mitigate repercussions of the COVID-19 pandemic include tools that provide guidelines and interfaces to influence behavior, reduce exposure to the disease, and enable policy-driven avenues to return to a sense of normalcy. This paper presents the implementation and early evaluation of a return-to-work COVID-19 symptom and risk assessment tool. The system was implemented across 34 institutions of health and education in Alabama, including more than 174,000 users with over 4 million total uses and more than 86,000 reports of exposure risk between July 2020 and April 2021.

**Objective:**

This study aimed to explore the usage of technology, specifically a COVID-19 symptom and risk assessment tool, to mitigate exposure to COVID-19 within public spaces. More specifically, the objective was to assess the relationship between user-reported symptoms and exposure via a mobile health app, with confirmed COVID-19 cases reported by the Alabama Department of Public Health (ADPH).

**Methods:**

This cross-sectional study evaluated the relationship between confirmed COVID-19 cases and user-reported COVID-19 symptoms and exposure reported through the Healthcheck web-based mobile application. A dependent variable for confirmed COVID-19 cases in Alabama was obtained from ADPH. Independent variables (ie, health symptoms and exposure) were collected through Healthcheck survey data and included measures assessing COVID-19–related risk levels and symptoms. Multiple linear regression was used to examine the relationship between ADPH-confirmed diagnosis of COVID-19 and self-reported health symptoms and exposure via Healthcheck that were analyzed across the state population but not connected at the individual patient level.

**Results:**

Regression analysis showed that the self-reported information collected by Healthcheck significantly affects the number of COVID-19–confirmed cases. The results demonstrate that the average number of confirmed COVID-19 cases increased by 5 (high risk: β=5.10; *P*=.001), decreased by 24 (sore throat: β=−24.03; *P*=.001), and increased by 21 (nausea or vomiting: β=21.67; *P*=.02) per day for every additional self-report of symptoms by Healthcheck survey respondents. Congestion or runny nose was the most frequently reported symptom. Sore throat, low risk, high risk, nausea, or vomiting were all statistically significant factors.

**Conclusions:**

The use of technology allowed organizations to remotely track a population as it is related to COVID-19. Healthcheck was a platform that aided in symptom tracking, risk assessment, and evaluation of status for admitting individuals into public spaces for people in the Alabama area. The confirmed relationship between symptom and exposure self-reporting using an app and population-wide confirmed cases suggests that further investigation is needed to determine the opportunity for such apps to mitigate disease spread at a community and individual level.

## Introduction

### Background

As of October 2022, there have been approximately 97 million COVID-19 cases, more than 1.1 million deaths in the United States, and about 7 million deaths worldwide [1]. The World Health Organization and the Centers for Disease Control and Prevention (CDC) emphasized continuous vigilance in preventing the spread of COVID-19 and its variants since they will continue to be a challenge on a global scale, even in an endemic environment [1,2].

The pandemic revealed vulnerabilities in our health care systems, motivating a push for disease mitigation and spurring several remote and digital care advancements. These include electronic tools for patients and health care providers to find innovative ways of coping with COVID-19 [3]. Technology solutions have addressed three conceptual components that were initially used in controlling and managing chronic diseases in the data-people-system framework by Bardhan et al [4]: (1) collection, derivation, integration, and articulation of health data; (2) interoperability of systems; and (3) guidelines and interfaces to guide people’s behavior, such as solutions that provided population screening, tracking infections, prioritizing the use and allocation of resources, and designing targeted responses. One important set of applications used globally, presented in this study, is a consumer-facing web-based/mobile symptom assessment tool. Little is known about the effectiveness and impacts of these tools on the population and the spread of the disease. Thus, this research investigates factors associated with symptom assessment tool use and reported COVID-19 symptoms and infections. We analyze a statewide initiative in Alabama, where a COVID-19 symptom assessment tool was implemented across K-12 and higher education institutions. Students, staff, and faculty were provided the tool to self-assess COVID-19 symptoms that calculated a risk level with actionable information and then presented the user with a green or red passport for entering campus events, such as class and athletic events. We explore the use of the Healthcheck symptom reporting app as a method to mitigate disease exposure in Alabama.

### Symptom Assessment Tools

The internet, including the web and mobile applications that operate on its infrastructure, has become integral to public health surveillance [5]. Maintaining access to school, work, and events has often meant that certain checkpoints have been put in place to mitigate disease exposure, and thus the spread of infection [6]. Such measures, including quarantining, regular testing, vaccination, and face masking, have been combined with digital symptom assessment tools, temperature checks, and the use of hand sanitizer upon entering an establishment [6,7]. Digital symptom assessment tools have become an integral method meant to help individuals: self-assess symptoms; provide daily prompts that query for symptom updates; and, with the help of built-in guidance, help self-triage. These apps collect data from individuals on risk factors (eg, age and comorbidities), symptoms, clinical outcomes, disease exposure, and geographical hot spots to inform health agencies and the public [8]. If the user obtains a result that could indicate a COVID-19 exposure or infection, some tools may provide actionable guidance specific to the user and the symptoms indicated. These tools may be combined with contact tracing or exposure notification and travel, work, or event passport functionality [9,10]. These tools have been most commonly presented as survey-oriented web and mobile applications [11] or as interactive chatbots [12], with some relying on artificial intelligence capabilities [13].

### Use

Symptom assessment tools have been implemented and used broadly [11] and globally [14-16]. These tools have been offered by government agencies and private institutions alike, largely for free public use [9,14,17]. Many organizations used COVID-19 symptom assessment tools as a survey or interactive chatbots, and some with “passports” to display the results of the symptom assessment tool and provide access to work or school facilities [18]. These “passport” tools generally promote or require daily completion before entering the place of work, study, or care center. A compliance report informs the employer or administrator of the completed survey. The person would not be allowed to enter the premises unless the survey, which may be particular to each workplace, is conducted and then yields a green passport, indicating safe entry. Typically, the green passport would be displayed for ready visualization by anyone checking.

### Benefits

Various models around symptom perception or self-care monitoring for chronic diseases suggest a link between information input (symptoms awareness) and behavior (seeking medical care) [19]. The framing of these models places symptom management in the hands of the users. With communicable diseases, the timing for symptom reporting is critical as the results disallow people from entering crowded spaces with symptoms. Certain suggestions or required behaviors, such as isolation, are then enacted at home [20]. The benefits of symptom assessment tools for nonchronic disease states, however, have largely been proposed by researchers with little empirical evidence of their impacts to date [21]. Possible benefits include the ability to forecast new outbreaks based on the data collected [22], more efficient alerting and isolating high-risk individuals and thereby preventing or reducing new infections, and improving how information is communicated to users [23,24]. Reported benefits include their ability to facilitate triaging large international and dispersed populations of patients seeking health care services, which can also help with resource allocation [3].

Symptom assessment tools can aid in predicting a positive test verification [8], help identify patients at high risk of hospitalization [25], and increase symptom awareness and behavior change, especially as symptoms change over time [26]. Most COVID-19 symptom lists include fever or chills, cough, shortness of breath or difficulty breathing, fatigue, new muscle or body aches, headache, the new loss of taste or smell, sore throat, congestion or runny nose, nausea or vomiting, and diarrhea. Such symptoms can also be attributed to other ailments such as influenza or cold and therefore may be easily dismissed. When symptoms are easily dismissed, the risk of spread is greater because there is a lack of awareness of and behavior change with attributing those symptoms to COVID-19. Therefore, increasing the accessibility of a symptom assessment tool increases awareness of symptoms and the potential for behavior change [26].

More research is needed to build the body of evidence supporting these tools in a nonchronic disease environment and to establish guidelines for the most appropriate implementations, especially when time is of the essence, such as in the case of COVID-19. One study comparing the diagnostic accuracies of 10 web-based COVID-19 symptom assessment tools found high variability between them, with just 2 symptom assessment tools providing a good balance between sensitivity and specificity [11]. However, this same study has been criticized for comparing tools with different intended purposes, noting that the appropriateness of specific advice in a given situation is more important to the user than either specificity or sensitivity [27]. Yet, another study found that general practitioners performed better with condition-suggestion accuracy than symptom assessment apps but also found that all three metrics (coverage, condition accuracy, and urgency advice) significantly varied across symptom assessment apps [28].

### Challenges

Digital symptom assessment tools in the form of apps typically held on smartphones do pose challenges such as the correctness of a symptom assessment and the privacy of user information [8]. For the former issue, it was found that most symptom assessment tools differ in their correct assessment of COVID-19 control cases and that a balance between usability and clinical specificity is needed [11]. While digital symptom assessment tools may be more rapidly scalable than analog measures, data quality issues might also be affected since they are typically based on self-reported data [8]. Furthermore, there is the potential for patient-led assessment tools to worsen outcomes by delaying appropriate clinical assessment [17]. In addition, many citizens may feel symptom assessment (whether analog or digital) violates privacy [29,30]. Lastly, symptom assessment tools can lead to overreporting of symptoms [26]. Governments or other institutions may mandate the use of such a tool and have done so in various settings worldwide. For example, in India, the home ministry mandated that all public or private workers use a government-backed COVID-19 tracking app called Aarogya Setu [15]. Even when government institutions do not require such technologies, employers and organizations might require them, leaving workers feeling their autonomy and privacy have been breached [3,29]. Finally, the benefits of symptom assessment tools may depend on many mediating factors, such as availability and access to the technology (smartphone), economics (cost) of using the technology, and public trust in the technology and the government [31]. For example, an assessment of chatbot style assessment tools reported use by a younger demographic (mean 34.3, SD 14.4 years), raising concerns that the most vulnerable population of older citizens may be too difficult to access [16,32,33].

### Use in Education and Work Environments

In the education environment, many schools and universities developed a variety of surveys meant for symptom assessment. Most of these were confined to that local environment; in other words, they were developed by that school for their use, and many were paper based. The CDC’s Coronavirus Self-Checker is available on their website and as a customizable widget that health departments and health care systems could add to their website. Other companies have also built apps that can be used for screening, tracking, and providing notifications for schools.

In Alabama, researchers responded early to the need for a symptom assessment tool to keep schools open. The response resulted in Healthcheck. Healthcheck is a COVID-19 symptom assessment web-based application that is accessible by computer or smartphone. The overarching goal of Healthcheck was to provide a platform for work or school re-entry for educational institutions and to serve as the cornerstone for the state of Alabama’s commitment to support education. Healthcheck includes a symptom assessment tool, a risk assessment algorithm with organization-specific actionable guidance, and a passport for entry onto campus and into events, such as sporting events. Healthcheck was considered 1 integral component of a comprehensive work or school re-entry plan inclusive of virus testing, education and communication about safe behaviors, social distancing, symptom checking, and exposure notification. This paper reports on the Healthcheck implementation and explores how usage of the COVID-19 symptom and risk assessment tool may have affected COVID-19 cases across Alabama.

### App Design, Development, and Implementation

#### Design

Drawing from CDC guidelines for COVID-19, a set of guiding principles for Healthcheck were determined using an iterative design process [34,35] to interview and gain feedback from health informatics experts, university senior administrators, and public health and infectious disease experts across functional units at a major medical university in the southeastern United States. The resulting requirements were used to develop Healthcheck. A 2-week pilot of approximately 200 research staff was conducted at the University of Alabama at Birmingham in May 2020. Pilot participants provided voluntary feedback throughout the 2 weeks, and issues were prioritized by the team for inclusion in the next version of Healthcheck. During the pilot test, Healthcheck’s accessibility, reliability, and accuracy were assessed, and its performance, usability, and security were improved based on user feedback. In June 2020, Healthcheck went live on one campus with phased rollouts across the state.

To mitigate privacy concerns for Healthcheck, it was designed and implemented to function in a single sign-on (SSO) environment. This meant that users signed on to their school network, and a token was passed to Healthcheck for secure access and use. As a web-based application, all appropriate security certificates were maintained and updated throughout the project.

#### Development

##### Overview

The Healthcheck passport tool was envisioned to provide a risk assessment and report for returning to work or school. The final screen of Healthcheck thus provides a green indicator for “clear to enter” (eg, green) or red for “not clear to enter” to be used for campus and sporting event entrance. Risk level calculations were determined by researchers and medical staff as follows.

##### Risk Level 3 Calculation Method: Red

Those users whose Healthcheck selections match the below criteria receive risk level 3. The user has been in isolation; the user has been in contact with another person diagnosed or under investigation for COVID-19; and the user has one of the following COVID-19 symptoms: fever, cough, difficulty breathing, and loss of taste or smell.

##### Risk Level 2 Calculation Method: Yellow

Those users whose Healthcheck selections match the below criteria receive risk level 2: user reports 2 symptoms other than fever, cough, difficulty breathing, loss of taste or smell, or none.

##### Risk Level 1 Calculation Method: Green

Users whose Healthcheck selections include items other than those described for risk levels 2 and 3 fall into this category.

Each passport would need to deliver individual actionable guidance specific for the school or organization to which it applied. While each school or organization could set its minimum compliance standard, it was envisioned that users would complete Healthcheck daily, including weekends and holidays, if campus attendance would be needed. For those who would be exempt from being on campus (ie, remote workers), Healthcheck would not be required.

The digital format of Healthcheck would need to enable updates in a continuously changing environment, which may also mean a change in protocols for screening as informed by health authorities. In addition to being flexible, this format would need to be a scalable option that helps organizations provide a user-centered solution to their safety needs.

After completing Healthcheck activity, workers would receive a digital badge that clears them for the day. This passport would need to be displayed at access control points that notify employers. The workers that do not pass this clearing would be instructed to quarantine at home and be provided with advice on self-care, monitoring, testing, or additional care. This type of work passport would have the dual purpose of protecting everyone at the workplace and providing checks and care on an individual basis.

Healthcheck was designed to include 5 primary screens. The first screen is the log-in screen that authenticates with an organization’s central authentication system.

**Figure 1:**
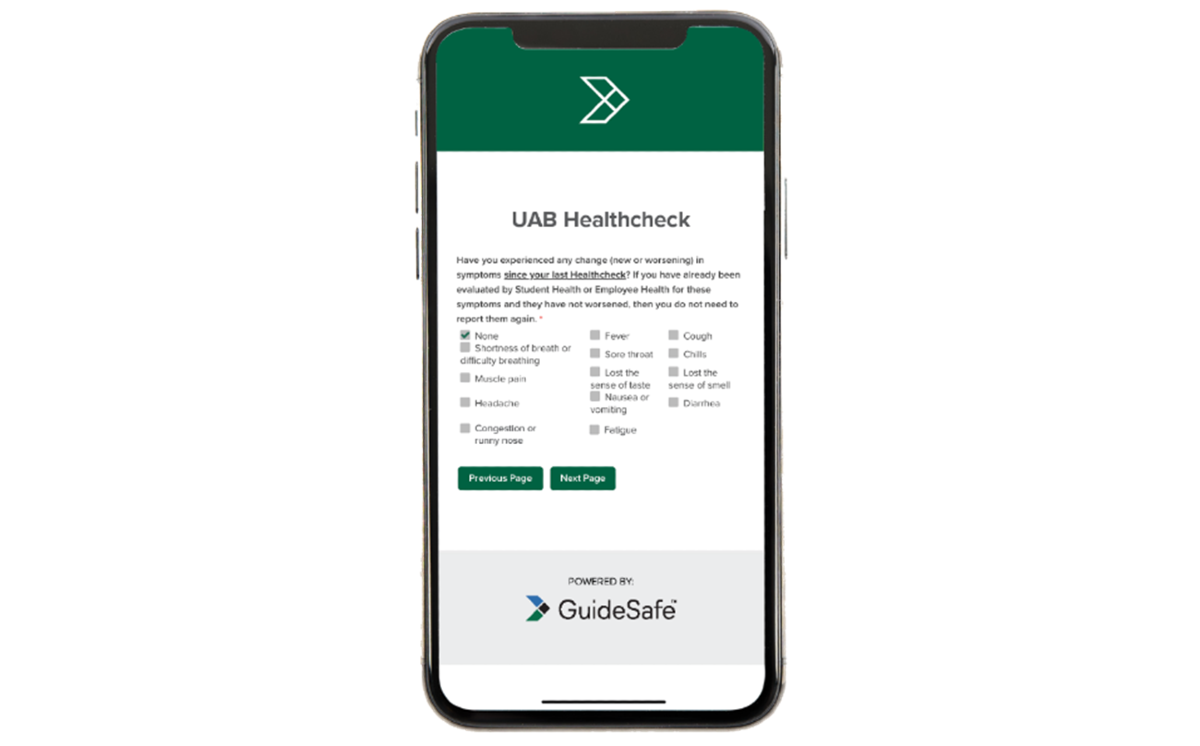
Screen 2—Healthcheck COVID-19 symptom assessment.

**Figure 2:**
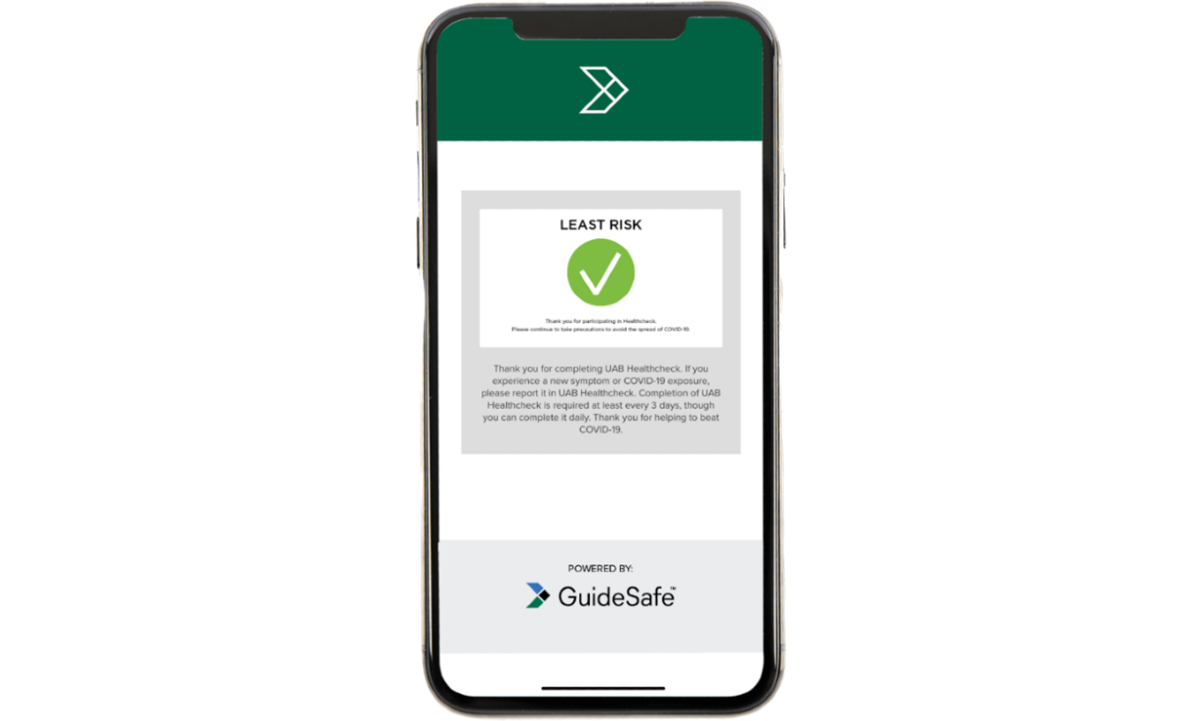
Screen 5—Healthcheck: COVID-19 risk level reporter.

The second screen (Figure 1) checks for COVID-19 symptoms as per CDC guidelines, while the next screen asks the user to report if they have been in contact with another person who was diagnosed or is under investigation for COVID-19 since the user’s last Healthcheck report. The next screen asks whether the user has been quarantined or recommended to be in quarantine, while the final screen (Figure 2) displays the user’s risk level. If users show low risk, they are able to attend work or school subject to any other additional screenings or health care provider recommendations.

### Implementation

Healthcheck went through a phased implementation over an approximately 6-week period in schools across the state. Implementation involved 3 elements: communication, technical implementation, and compliance reporting. In terms of communication, we had a communications toolkit that we supplied to the schools such that they only needed to insert information about their school, such as contact people and school colors. Because Healthcheck was designed for SSO capability, and most schools had an existing SSO environment, technical implementation was somewhat streamlined. During the implementation process, we found that many schools used Google Schools, and our technical team would help them set up the SSO included in Google Schools. Lastly, compliance reporting involved setting up a secure and encrypted method of transport for the Healthcheck compliance reports, which were sent to schools hourly.

## Methods

### Data Collection

A cross-sectional study examined the relationship between confirmed COVID-19 cases and COVID-19–related symptoms and exposure reported through Healthcheck. The data used in this study included 1 dependent variable and 16 independent variables. The dependent variable is the confirmed COVID-19 cases in Alabama as reported by the Alabama Department of Public Health (ADPH), while the independent variables are health symptoms collected by Healthcheck.

Healthcheck consists of questions that assess symptoms and exposure to COVID-19 and was designed for minimal data input by the user and maximum information output by the application. Healthcheck surveys could be completed only daily by each unique user. Although faculty, students, and staff were encouraged to participate, enforcement of daily use was up to the discretion of each educational institution. The initial setup for first-time users to use Healthcheck takes approximately 20 seconds. Each subsequent survey takes approximately 5 seconds to complete. Users are instructed to only report *new* symptoms that are not related to existing health conditions or recent activity (eg, seasonal allergies). Deidentified data on 545,887 confirmed COVID-19 cases were obtained from the ADPH.

The primary outcome of interest was daily confirmed COVID-19 cases. All laboratories in Alabama are required to report within 4 hours all confirmed or probable cases to ADPH [2], in which a probable case is a case that has compatible COVID-19 symptoms with either an epidemiological link to a laboratory-confirmed case or a member of a risk cohort as defined by public health authorities during an outbreak. Once reported to ADPH, a case status of confirmed is assigned based on case ADPH COVID-19 case definition. Independent variables of interest in the Healthcheck survey data included measures assessing COVID-19–related risk levels and symptoms. Survey respondents were asked to indicate if they currently were experiencing 1 or more of the following symptoms (yes or no): shortness of breath, muscle pain, loss of sense of taste, loss of sense of smell, diarrhea, chills, cough, congestion or runny nose, sore throat, nausea or vomiting, headache, fever, fatigue, and other. For determining how the algorithm classified the risk, we consulted with communicable disease experts at our ADPH, epidemiologists, and infectious disease experts. Risk level was categorized into 3 risk categories: high risk, may be at risk, and low risk. Risk, in this context, was the risk level for having COVID-19. Survey respondents were assigned a status of *high risk* if they were exposed to a known case, shortness of breath, cough, loss of smell, loss of taste, or fever. Elevated temperatures that increased from the previous temperature or were greater than 38.8 °C were considered to be high risk. It was determined that if the temperature is maintained without other COVID-19–related symptoms, the person is likely seeking medical care or has something else (flu, etc). Survey respondents were assigned a status of *may be at risk* if they had 2 or more of the 14 symptoms. *Low-risk* status was assigned if the respondent did not meet the criteria for *high risk* or *may be at risk.*

### Exclusion Criteria

Various exclusion criteria were applied to prepare the data for modeling. The criteria are (1) ADPH cases with missing or invalid zip codes were excluded; (2) Healthcheck survey observations from November 18, 2020, through January 7, 2021, were excluded because some colleges and universities elected to hold classes for the remainder of the Fall 2020 semester remotely or school was not in session due to holiday break; (3) ADPH case data from March 2020 through June 2020 and from May 2021 through June 2021 were excluded because Healthcheck survey data did not contain observations for those months. The final sample contained 312,504 COVID-19 cases and 4,336,472 survey entries from 22 colleges and universities and 13 K-12 organizations.

### Ethics Approval

This study, using deidentified data, was reviewed by the institutional review board (IRB) of the University of Alabama at Birmingham and classified as nonhuman subjects research (IRB-300010983). While case-level cross-verification between Healthcheck users and confirmed COVID-19 cases would have provided stronger validity to this study, identification of study participants was not permitted.

### Data Analysis

The initial data set encompassed the reported COVID-19 cases throughout each day, amounting to a total of 312,504 cases. Subsequently, the cases were consolidated on a daily basis, resulting in each observation representing the cumulative count of cases per day. The 4,336,472 entries in the Healthcheck survey were aggregated by day, with each observation containing the total number of entries per day. The aggregated daily COVID-19 cases were linked to the aggregated daily Healthcheck entries. For example, each day’s observation included how many confirmed COVID-19 cases (dependent variable) and the frequency of symptoms for all patients (independent variables). In other words, all observations are aggregated on a daily level. The final data set contained 247 observation days from July 2020 through April 2021.

Multiple linear regression was used to estimate the relationship between patients with a confirmed diagnosis of COVID-19 and self-reported symptoms and exposure via Healthcheck. Multiple linear regression models examined the relationship between 1 dependent variable and more than 1 independent variable (equation 1), where *Y* is the dependent variable, (*X*_1_, *X*_2_, … *X_m_*) are the independent variables, *β*_0_ is the intercept, (*β*_1_, *β*_2_,… *β_m_*) are the coefficients of a regression model, *m* is the number of independent variables, and *ε* is the random error term. Standardized residuals from the linear regression were plotted to determine if the error term was normally distributed. Statistical analyses were performed using STATA (version 16; StataCorp) and Excel Data Analysis Regression tool software (Microsoft Corporation). The statistical significance level was set at *P*<.05. The multiple linear regression model is:

*Y* = *β*_0_ + *β*_1_*X*_1_ + *β*_1_*X*_1_ + … + *β_m_X_m_*
**(1)**

## Results

Table 1 presents study sample characteristics for the 4,336,472 total observation entries and 247 total observation days during the study period. The daily mean and median counts of confirmed COVID-19 cases were 1265.198 (SD 849.9001) and 1063 (IQR 114-5086), respectively. Congestion or runny nose was the most frequently reported symptom. Most survey respondents were in the low-risk category (n=4,267,267, 98.4%). No respondents met the may be at risk criteria. The Healthcheck user’s risk level, daily count of COVID-19 cases, and symptoms over the 247 observation days are presented in Multimedia Appendix 1.

**Table 1:** Characteristics of daily Healthcheck survey respondents.

Characteristics			Total observations (N=4,336,472), n (%)		Mean (SD)		Median (IQR)
Shortness of breath			5136 (0.12)		20.79 (22.89)		14 (1-141)
Muscle pain			9484 (0.22)		38.40 (37.29)		29 (0-255)
Loss of sense of taste			4519 (0.10)		18.30 (23.48)		12 (0-177)
Loss of sense of smell			5342 (0.12)		21.63 (27.18)		14 (0-199)
Diarrhea			7511 (0.17)		30.41 (29.31)		22 (0-179)
Chills			5624 (0.13)		22.77 (27.42)		16 (0-215)
Cough			17,504 (0.40)		70.87 (66.95)		52 (0-422)
Congestion or runny nose			36,929 (0.85)		149.51 (121.59)		115 (1-686)
Sore throat			20,035 (0.46)		81.11 (77.54)		58 (1-458)
Nausea or vomiting			6558 (0.15)		26.55 (2262)		20 (0-134)
Headache			26,017 (0.60)		105.33 (94.84)		77 (0-588)
Fever			5396 (0.12)		21.85 (21.56)		17 (0-166)
Fatigue			18,735 (0.43)		75.85 (71.50)		59 (0-451)
Other			99 (0.002)		0.40 (3.42)		0 (0-40)
**Risk level**							
	High risk	69,205 (1.60)		280.18 (261.33)		230 (2-1478)	
	Maybe at risk	0 (0)		0 (0)		0 (0)	
	Low risk	4,267,267 (98.4)		17,276.38 (9371.69)		17,442 (58-45,092)	

### Multiple Linear Regression

**Figure 3:**
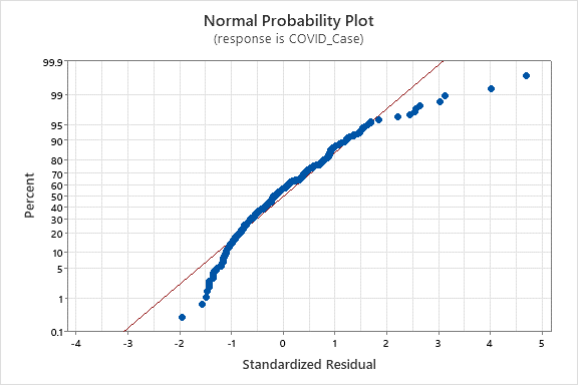
Normality plot.

Figure 3 presents the normality plot, indicating the residuals are normally distributed, satisfying the normality assumption of linear regression. Table 2 presents the results of the multiple linear regression model. It can be noted that the *R*^2^ and the *R*^2^ adjusted are 29% (*R*^2^=0.2907) and 24% (*R*^2^=0.2413), respectively. This means that about 29% of the variance in the daily count of confirmed COVID-19 cases can be explained by the independent variables in the regression model, which are collected by Healthcheck that we created for the study. Although the *R*^2^ and the *R*^2^ adjusted are relatively low, achieving about 29% of the variance using Healthcheck can be acceptable. The reason is that there are many variables affecting COVID-19 that Healthcheck cannot collect, including medical conditions, cultural awareness, environment, and so forth.

**Table 2:** Multiple linear regression results.

	Coefficient	SE	*T* statistic	*P* value	Lower 95%	Upper 95%
Low risk	−0.04	0.01	−2.56	.01^a^	−0.07	−0.01
High risk	5.10	1.13	4.50	.001^a^	2.87	7.33
Shortness of breath	−20.10	12.00	−1.68	.10^b^	−43.73	3.54
Muscle pain	13.80	8.51	1.62	.11	−2.96	30.56
Loss of sense of taste	−6.64	17.27	−0.38	.70	−40.66	27.37
Loss of sense of smell	−1.62	15.61	−0.10	.92	−32.39	29.15
Diarrhea	−2.59	7.91	−0.33	.74	−18.17	12.99
Chills	−9.80	11.03	−0.89	.38	−31.53	11.93
Cough	8.91	6.70	1.33	.18	−4.29	22.10
Congestion or runny nose	3.99	2.96	1.35	.18	−1.84	9.81
Sore throat	−24.03	4.57	−5.26	.001^a^	−33.03	−15.03
Nausea or vomiting	21.67	9.13	2.37	.02^a^	3.67	39.67
Headache	3.81	4.31	0.88	.38	−4.68	12.31
Fever	6.46	11.94	0.54	.59	−17.08	29.99
Fatigue	−8.81	6.56	−1.34	.18	−21.73	4.12
Other	23.23	14.32	1.62	.11	−4.99	51.45

^a^Coefficients with a significance level of *P*<.05.

^b^Coefficients with a significance level of *P*<.10.

The variables low risk, high risk, sore throat, and nausea or vomiting were statistically significant. For every additional self-report of symptoms by Healthcheck survey respondents, the average number of confirmed COVID-19 cases increased by 5 (high risk: β=5.10; *P*=.001), decreased by 24 (sore throat: β=−24.03; *P*=.001), and increased by 21 (nausea or vomiting: β=21.67; *P*=.02) per day.

## Discussion

### Principal Findings

This paper describes the implementation and early evaluation of Healthcheck, a COVID-19 symptom assessment web-based app used in Alabama during the COVID-19 outbreak. Healthcheck was made broadly available to help organizations track COVID-19 symptoms and facilitate return to school activities while balancing stakeholder needs. Healthcheck was used by more than 174,000 users, providing a focused population-wide evaluation. COVID-19 symptom assessment and risk assessment tools, as the one reported in this study, eventually became more commonplace across the globe to mitigate the pandemic [8,36,37].

Our study showed a positive relationship between survey respondents who reported nausea or vomiting and an increased number of confirmed COVID-19 cases in the general public. It is important to note that individuals can exhibit gastrointestinal symptoms, including nausea or vomiting as the first clinical manifestation of COVID-19 as shown by several studies [38-40]. Another finding from our study was the negative relationship between a sore throat and the increased number of confirmed COVID-19 cases, although there has been mixed literature on sore throat as a symptom of COVID-19 [38,41]. A sore throat could also be a symptom of many other conditions such as seasonal allergies [42]. Sore throat has previously been negatively correlated with COVID-19 infection [43]. Our study also showed a positive relationship between the increased number of confirmed COVID-19 cases in the general public in Alabama and survey respondents who exhibited more high-risk characteristics based on the self-reported information logged into Healthcheck. The opposite was the case for survey respondents who exhibited low-risk characteristics. Other studies have shown strong correlations between COVID-19–confirmed cases and symptoms, including malaise, fatigue, headache, cough, fever, dysgeusia, dyspnea, sputum, and hyposmia [43,44]. While some studies have shown that COVID-19 symptom trackers vary widely in predictions [8,11,16], we believe these apps provide imperfect yet some valuable performing indicators, as illustrated by our study.

There are some disadvantages with apps like Healthcheck as seen in other studies, such as issues with data quality since symptoms are self-reported [8], over/underreporting symptoms [8,26], delay in appropriate clinical assessment, [17] and individuals feeling that their privacy has been violated [8,30]. It is also important to point out that relying on digital technology, such as Healthcheck, can highlight socioeconomic inequalities, further contributing to health care disparities [45,46]. For this reason, it was important to develop Healthcheck as a web-based app to reach a wider audience rather than a pure mobile app.

One of the benefits of the Healthcheck, particularly during COVID-19, when the CDC advocated home symptom screening of all school students rather than standard in-person COVID-19 symptom screening, was that it was able to eliminate the need for organizations to have personnel in facilities screening for COVID-19 symptoms. This helped organizations reduce frequent and close contact between people and mitigate the risk of COVID-19 exposure while also following the CDC guidelines for public health safety. The acceptability of apps of this nature depends on how the results are intended to be used. Even though apps like Healthcheck cannot replace a COVID-19 test, they do have the capability of forecasting new outbreaks based. The data collected by Healthcheck can be used to develop deep learning time series models to predict outbreaks at the individual or aggregated (ie, daily and monthly) levels. This will help in alerting organizations of high-risk individuals, preventing or reducing new infections, and improving how information is communicated to users [22-24,47]. In the event of another pandemic or disease outbreak, the capability of such apps can help stakeholders and organizations plan and mitigate the risk of disease spread.

### Limitations

The limitations of symptom assessment apps like Healthcheck are their inability to detect presymptomatic or asymptomatic infected individuals unless combined with data providing diagnostic results. They also rely on the ability of the user to report symptoms, usually without an assessment from a health care professional. Symptoms may take 2 to 14 days to develop after exposure to the virus [1]. Even early symptoms can sometimes be dismissed as general fatigue or seasonal allergies. On the other hand, since many symptoms of COVID-19 are present in other illnesses, including chronic medical conditions, some individuals may be counted as false positives and repeatedly excluded from work or school even though they do not have COVID-19 (or any other contagious illness). This is problematic as these employees and students may frequently miss work or school due to their medical conditions.

On the contrary, Healthcheck user bias could also play a potential role in limiting the study findings. Some users can have the intent to not report their actual symptoms as the resulting risk classification provides adequate justification to attend or not attend work or classes and thus bypass the purpose of symptom assessment. While case-level cross-verification of participants between Healthcheck users and confirmed COVID-19 cases could have mitigated these limitations and provided stronger validity to this study, identification of study participants was not permitted by IRB. Thus, due to the deidentification of the case data, it was impossible to know if Healthcheck users represented a similar distribution to the population in the state. This may represent a limitation in our analysis.

Although the sample size for this study is significant, limitations of these types of studies include the inability to analyze data based on a wider range of demographic, environmental, geographic, and health factors. The study was limited to 1 state in the southern United States. Nevertheless, the analysis provides important insights into the impact of such technologies on a large, geographically focused population. These limitations are responsible for the relatively low *R*^2^ value, which hinders the development of a better-fit model that can explain the variance in daily COVID-19–confirmed cases. Future research may evaluate these findings based on broad expert critical analysis.

### Conclusions

The Healthcheck system was implemented across 34 institutions of health and education in Alabama, including more than 174,000 users with over 4 million total uses between July 2020 and April 2021. The regression analysis results using this data set showed that the self-reported information collected by Healthcheck significantly affects the number of COVID-19–confirmed cases (as reported by ADPH) where the self-reporting occurred. Taking important study limitations into consideration, these results may suggest that when large groups of individuals self-report symptoms with an app, such as Healthcheck, there could be a mild to moderately significant effect on the total number of confirmed COVID-19 cases in regions where the self-reporting occurred. This paper represents an example of the further integration of health information technology into the daily lives of every individual during a unique worldwide pandemic. Technology-enabled remote health checking, risk assessment, and determination for work and school readiness or availability based on an individual's reported health or infection status have been represented in this paper and various settings across the globe as one population-level strategy for addressing a large-scale pandemic. Designing such tools that satisfy broad stakeholder groups is an ongoing design, implementation, and evaluation challenge.

## Appendix

Multimedia Appendix 1 User’s risk level and box and whisker plot of the total daily count over the 247 observation days in the study period.

## Abbreviations

ADPH: Alabama Department of Public Health
CDC: Centers for Disease Control and Prevention
IRB: institutional review board
SSO: single sign-on

## Data Availability

The institutional review board, as approved, does not allow for data sharing.
