# Supplementary material for: Predictors of COVID-19 From a Statewide Digital Symptom and Risk Assessment Tool: Cross-Sectional Study
Source: J Med Internet Res. 2023 Jul 25;25:e46026. doi: 10.2196/46026 (PMC10410382; doi:10.2196/46026)
Supplement: Multimedia Appendix 1 [file jmir_v25i1e46026_app1.pdf]

The column chart in Figure A.1 shows the user's risk level over the 247 observation days.

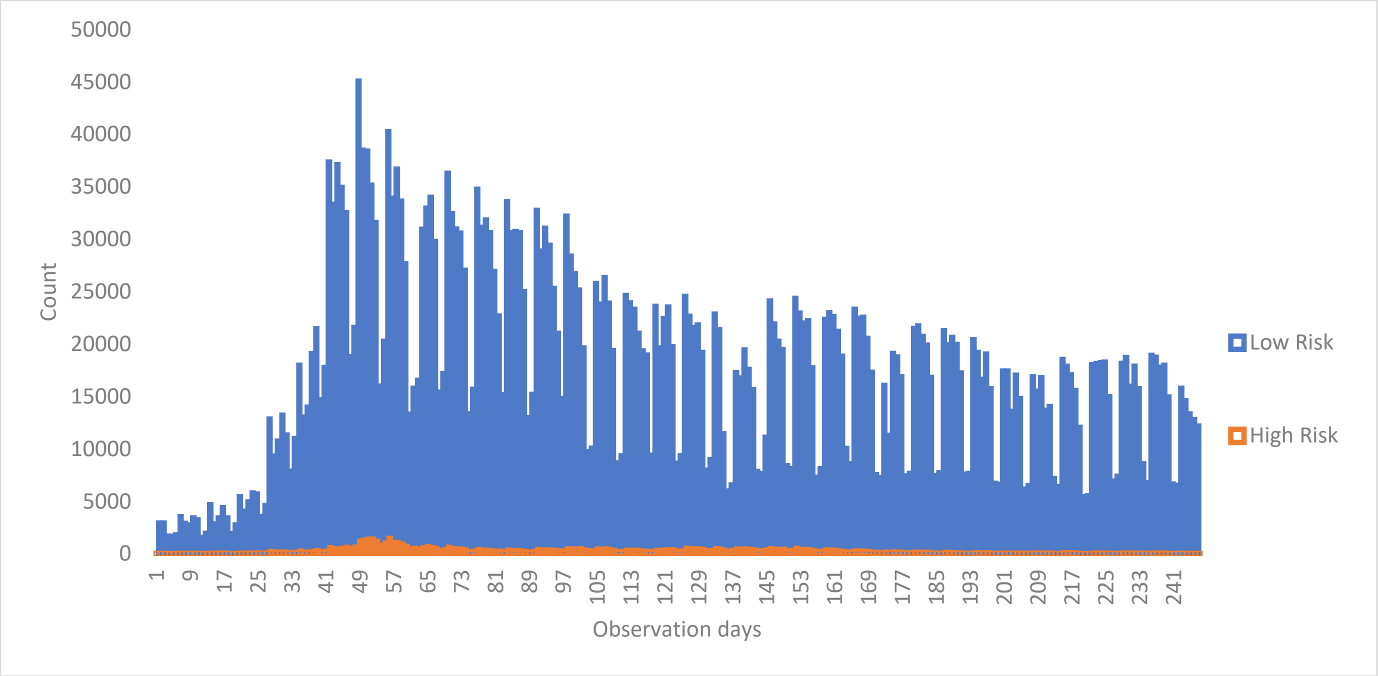

Figure A.1 User risk level

The box and whisker plots, Figures A.2 – A.16, (dependent variable and independent variables) shown below present the total daily count over the 247 observation days in the study period, highlighting quartiles, the mean, and outliers.

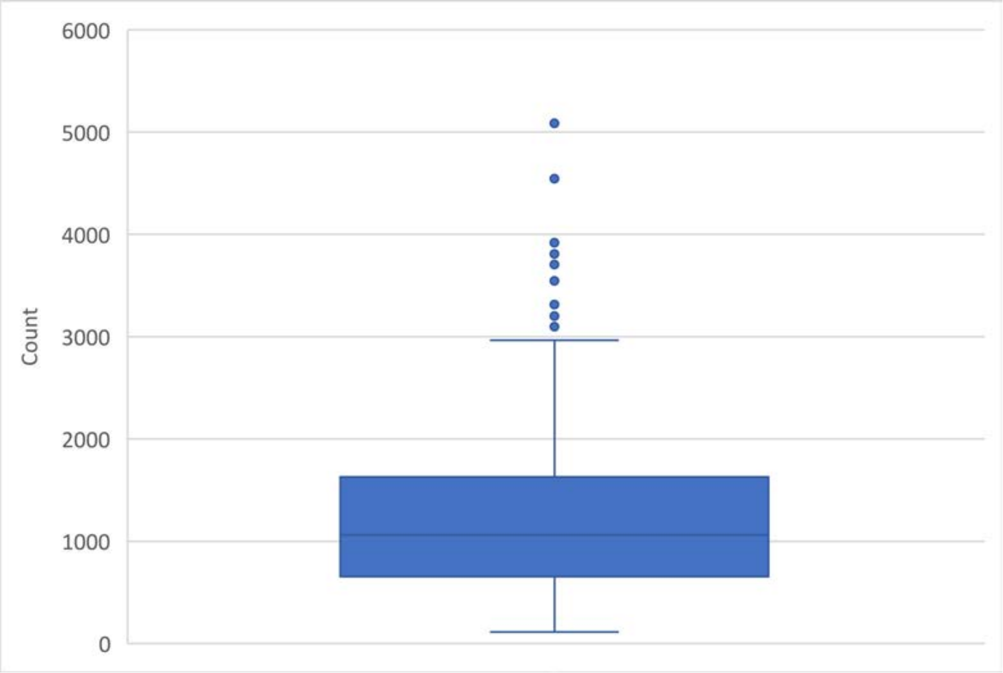

Figure A.2 Box and whisker plot of COVID-19 Cases

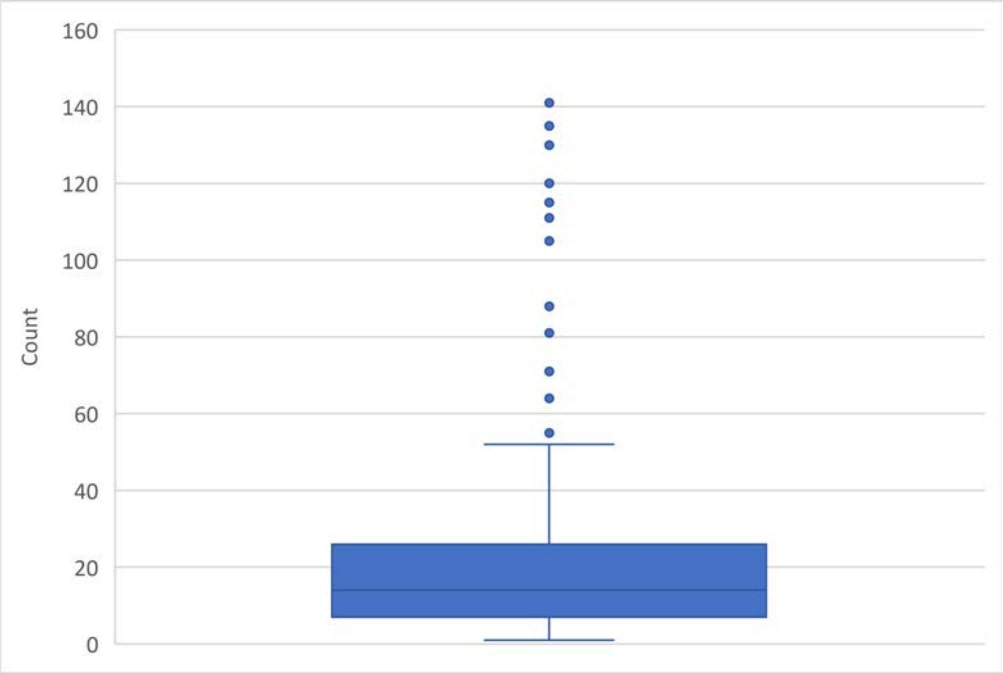

Figure A.3 Box and whisker plot of shortness of breath symptom

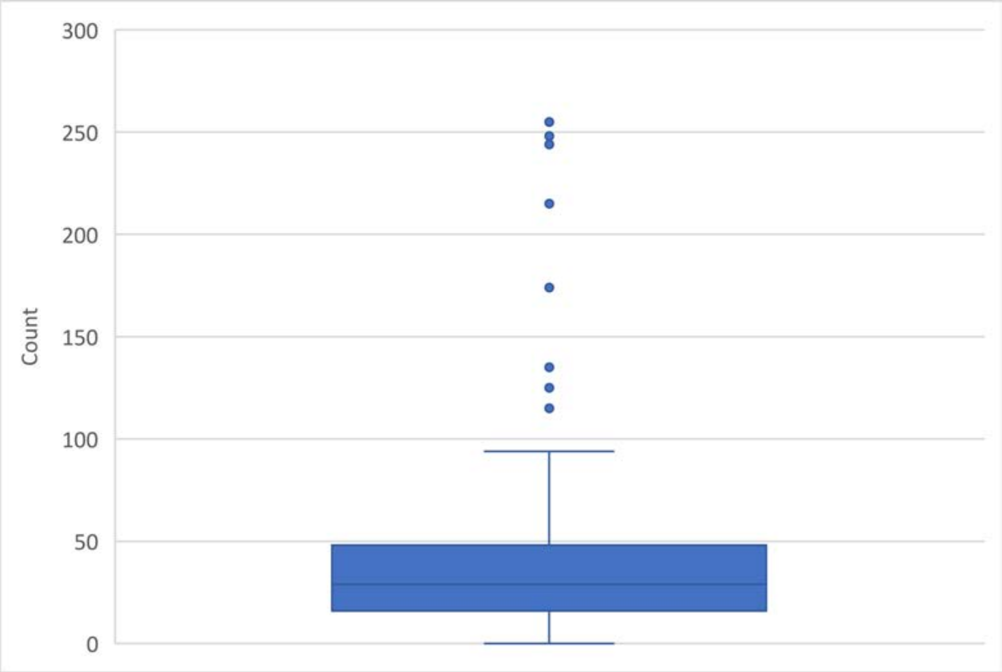

Figure A.4 Box and whisker plot of muscle pain symptom

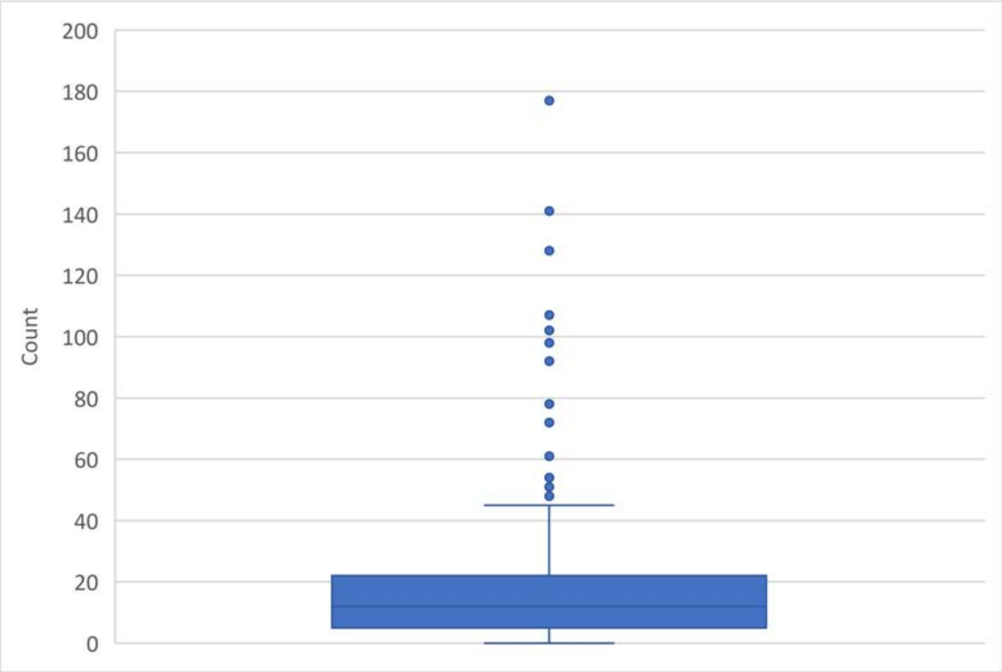

Figure A.5 Box and whisker plot of lost sense of taste symptom

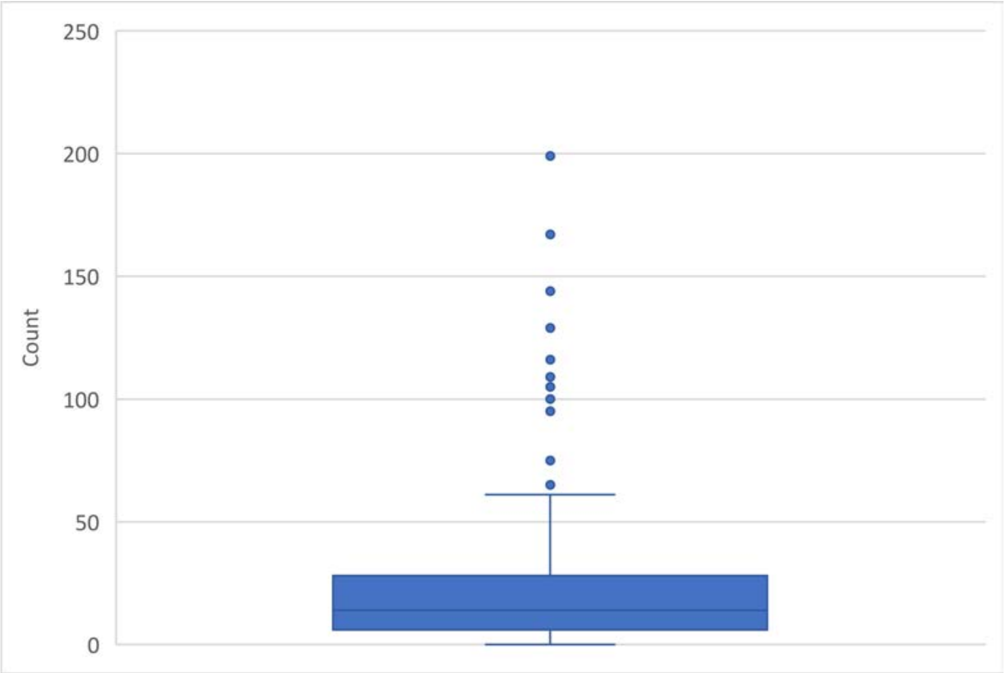

Figure A.6 Box and whisker plot of lost sense of smell symptom

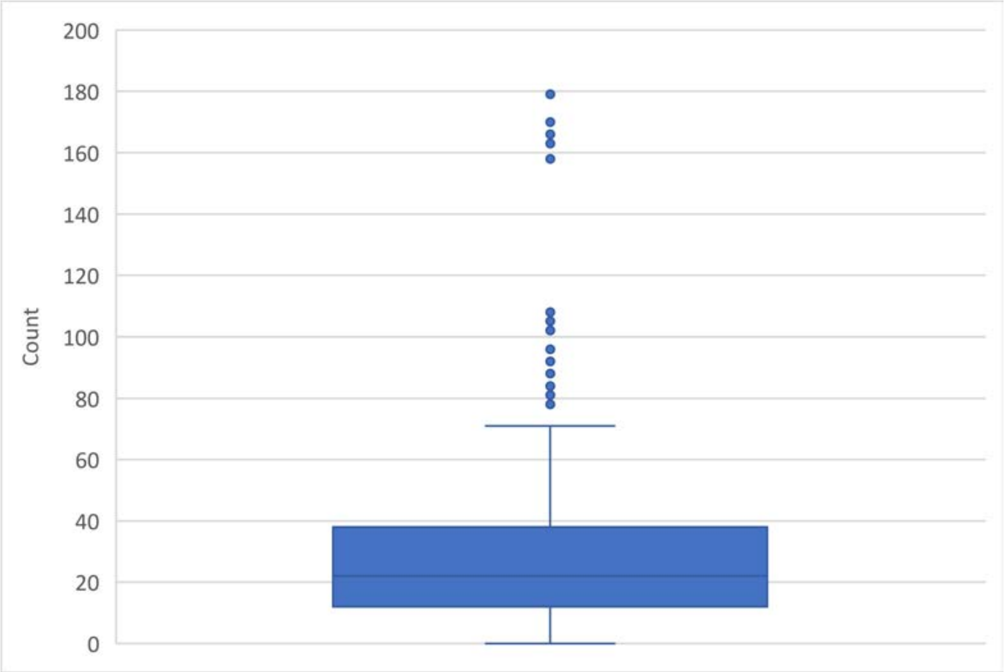

Figure A.7 Box and whisker plot of diarrhea symptom

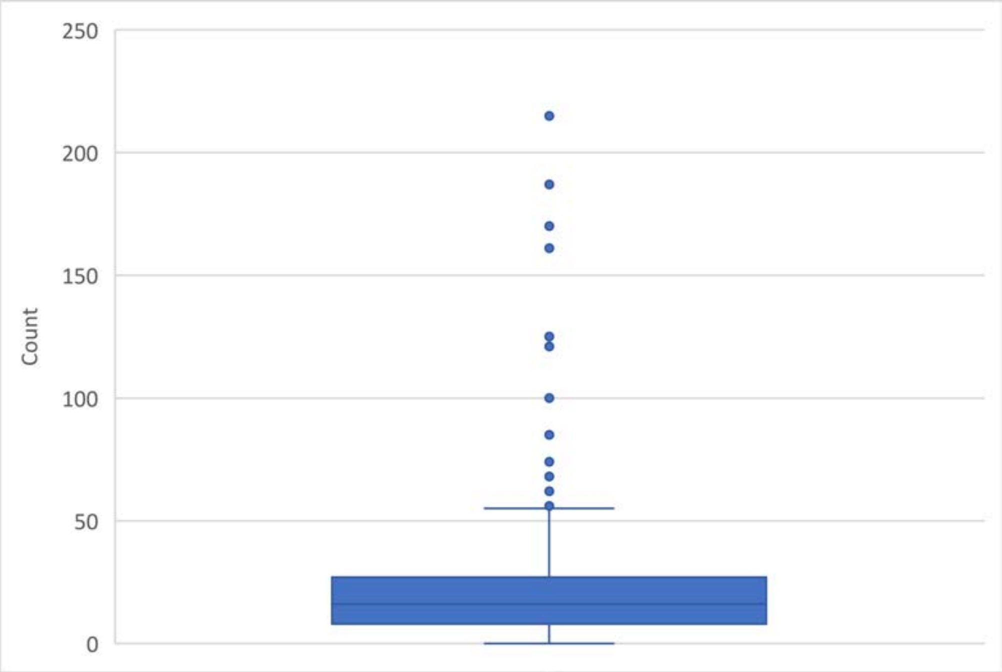

Figure A.8 Box and whisker plot of chills symptom

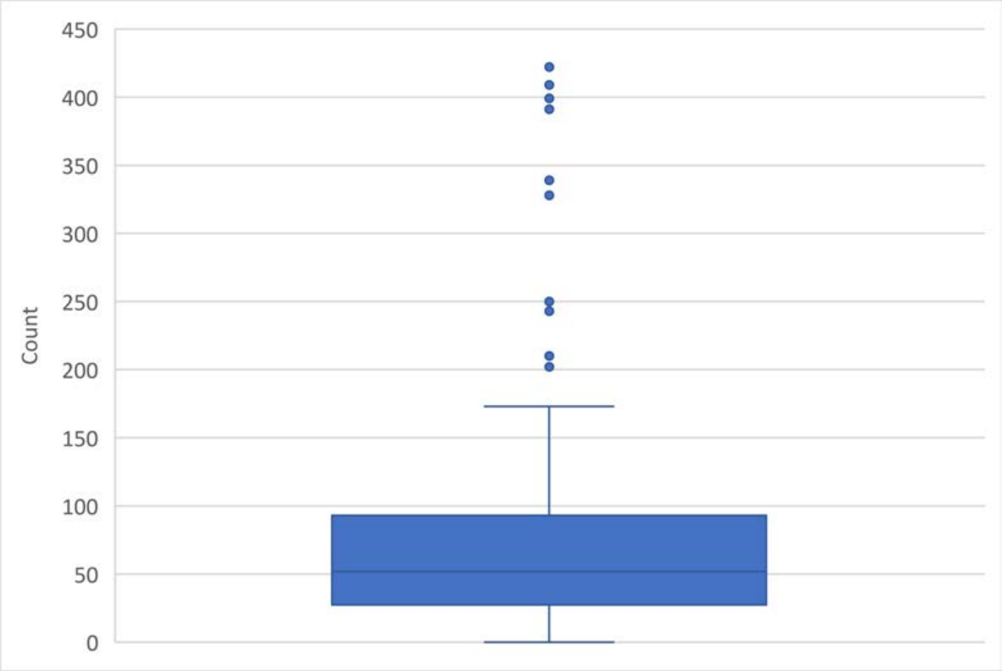

Figure A.9 Box and whisker plot of cough symptom

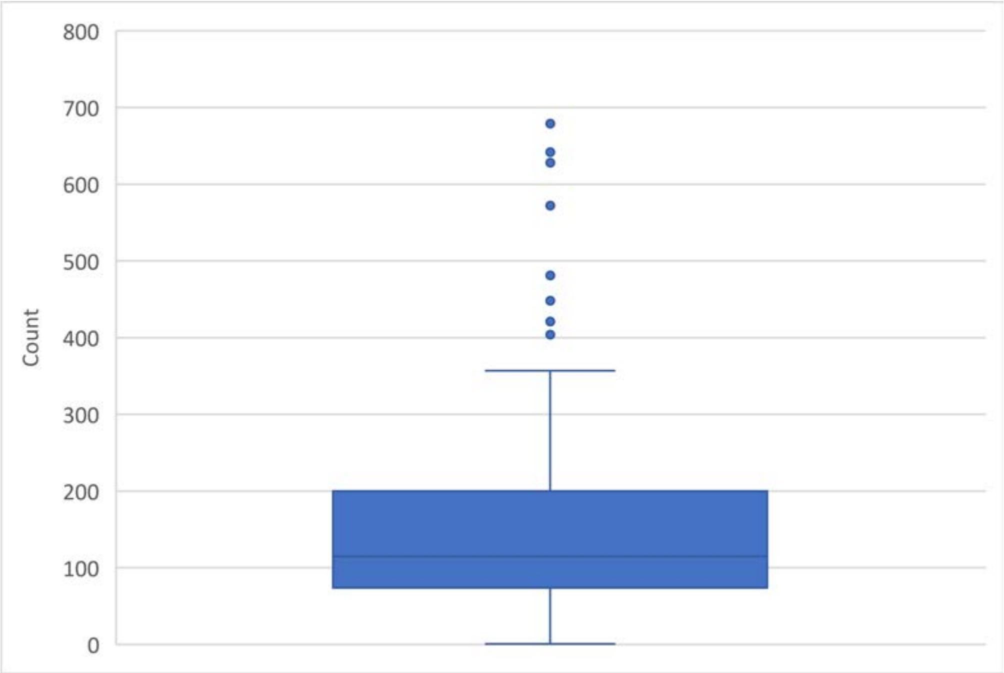

Figure A.10 Box and whisker plot of congestion or runny nose symptom

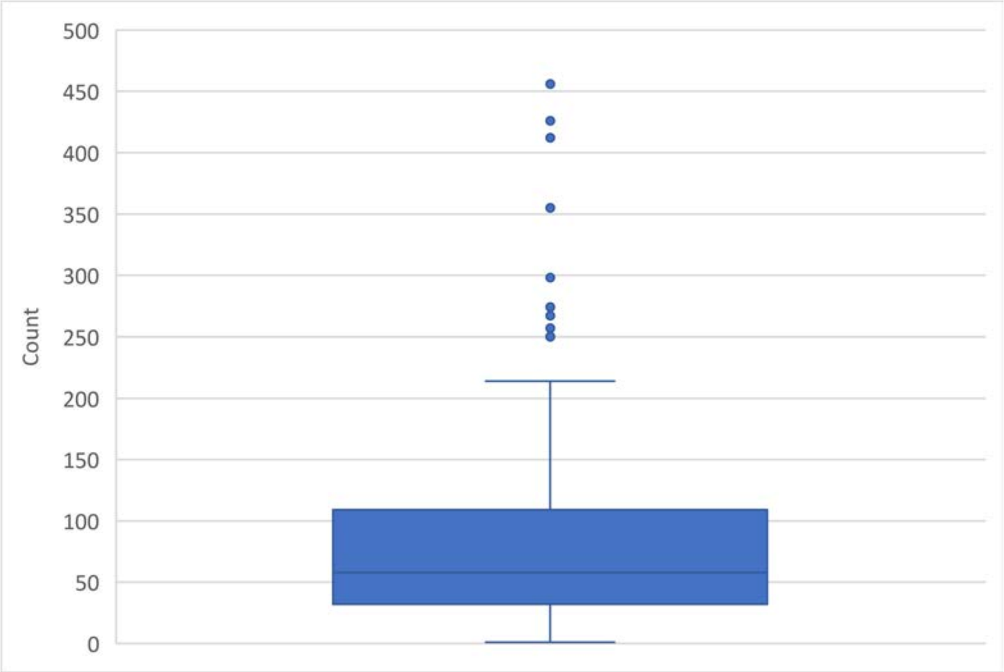

Figure A.11 Box and whisker plot of sore throat symptom

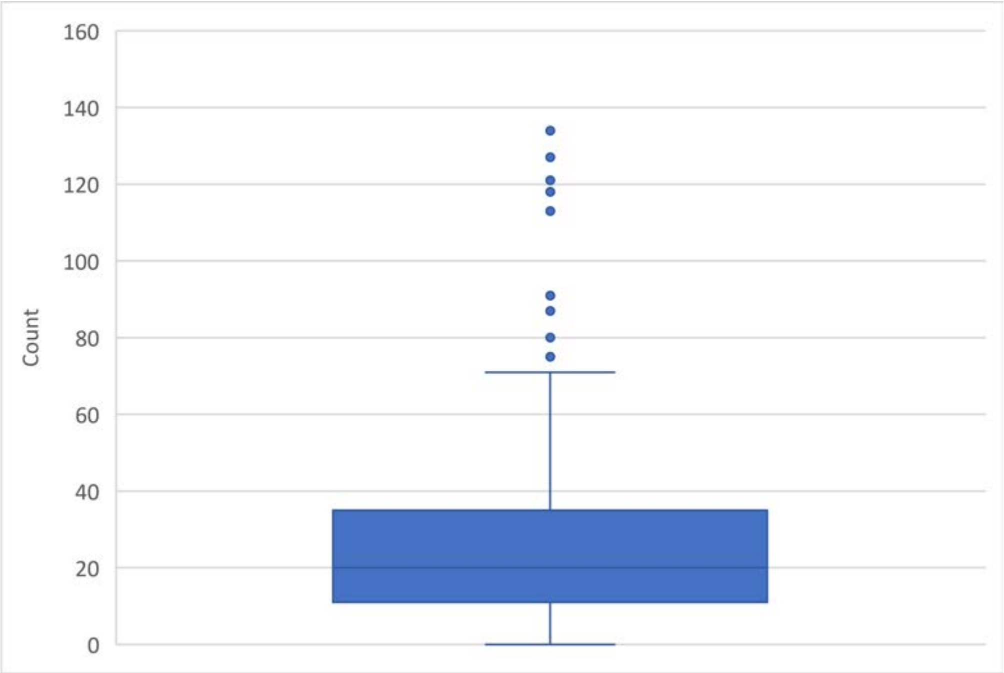

Figure A.12 Box and whisker plot of nausea or vomiting symptom

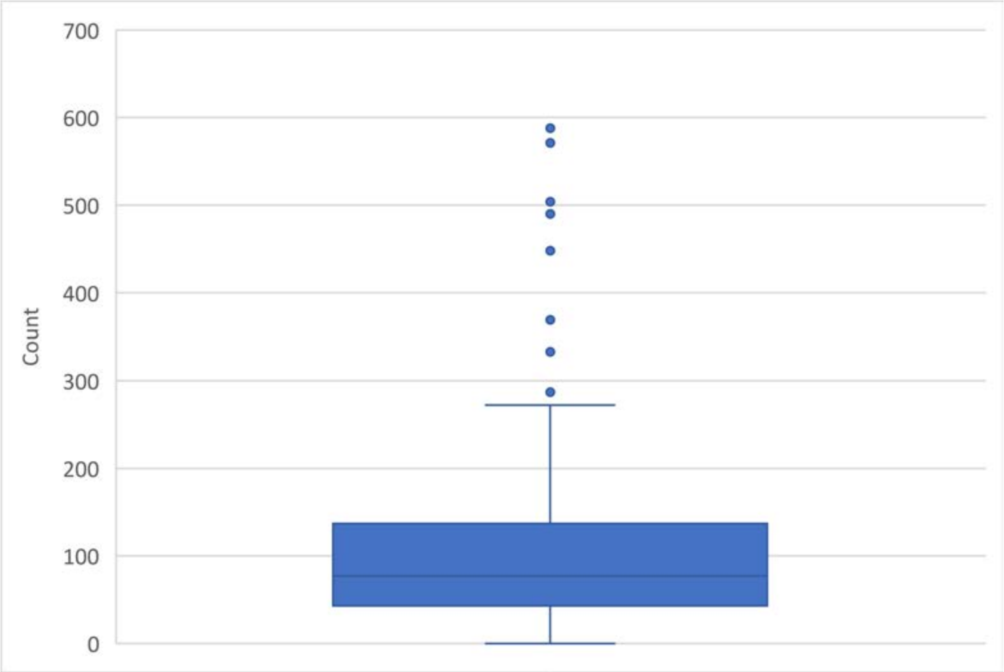

Figure A.13 Box and whisker plot of headache symptom

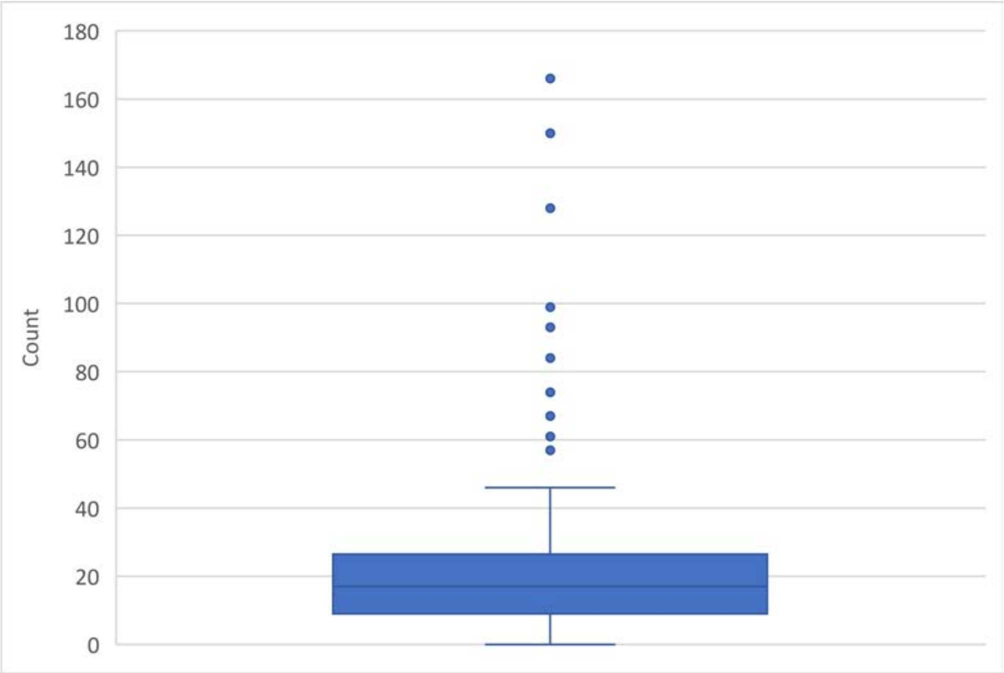

Figure A.14 Box and whisker plot of fever symptom

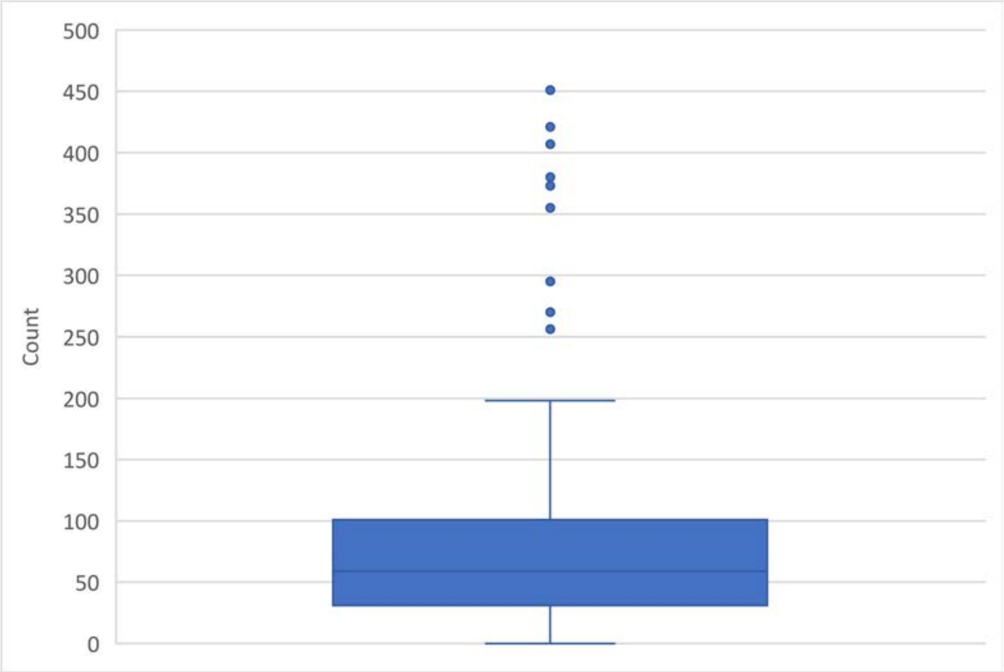

Figure A.15 Box and whisker plot of fatigue symptom

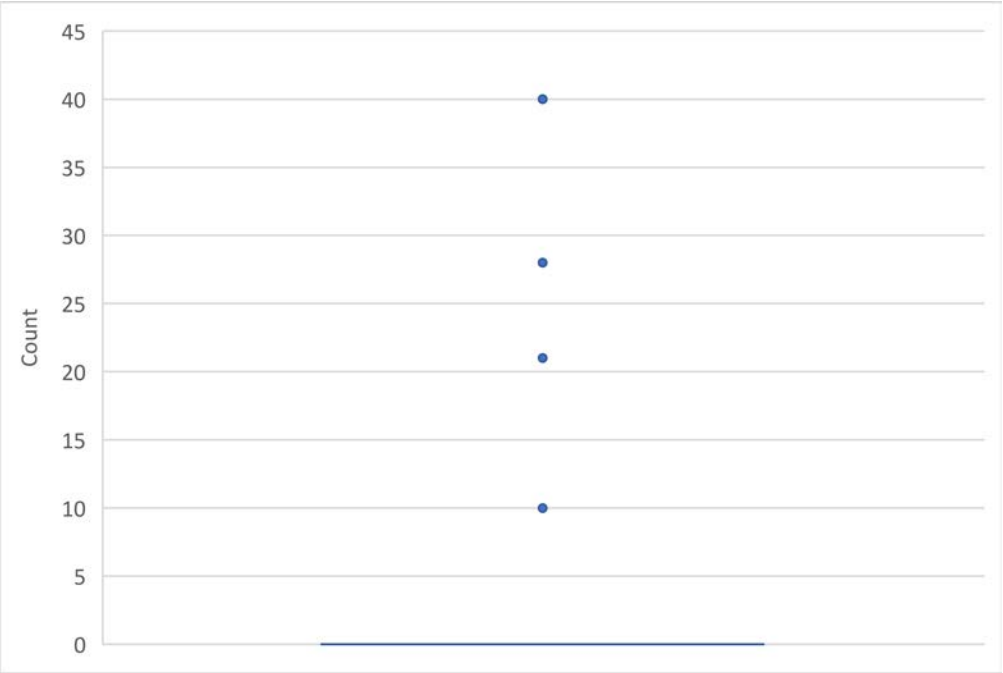

Figure A.16 Box and whisker plot of other symptoms
